# Integrated Network Pharmacology and Gut Microbiota Analysis Reveals the Alcoholic Extract of *Anacyclus pyrethrum* Root Prevents Nonalcoholic Fatty Liver Disease via the LPS/TLR4/NF-κB Pathway

**DOI:** 10.3390/ijms27104398

**Published:** 2026-05-14

**Authors:** Hao Yang, Lijuan Zhang, Xinle Tang

**Affiliations:** 1 School of Pharmacy, Xinjiang Medical University, Urumqi 830017, China; 15099334390@xjmu.edu.cn; 2 Xinjiang Key Laboratory of Uyghur Medical Research, Xinjiang Institute of Materia Medica, Urumqi 830004, China

**Keywords:** *Anacyclus pyrethrum* root ethanol extract, high-fat diet, glucose and lipid metabolism disorders, gut microbiota, non-alcoholic fatty liver disease

## Abstract

The global incidence of nonalcoholic fatty liver disease (NAFLD) is rising, with no approved pharmacotherapy available. Medicinal plants offer a potential preventive strategy. *Anacyclus pyrethrum* root exhibits anti-inflammatory and glucose-regulating properties, but its role in NAFLD prevention is unclear. This study aims to investigate the preventive effect of *Anacyclus pyrethrum* root ethanol extract (APE) against NAFLD and its underlying mechanisms. The chemical composition of APE was analyzed by UHPLC-HRMS. Network pharmacology predicted the potential signaling pathways underlying its protective effects against NAFLD. In a 12-week high-fat diet mice model, APE treatment led to measurements of blood glucose, lipid profiles, liver function parameters, histopathological changes in liver and colon, and gut microbiota alterations via 16S rDNA sequencing. In animal experiments, APE lowered fasting and random blood glucose, total cholesterol, triglycerides, LDL-C, AST, ALT, and serum lipopolysaccharide while increasing HDL-C, and alleviated hepatic steatosis. Network pharmacology suggested APE acts via TLR, NF-κB, and TNF pathways. In vivo, APE suppressed hepatic TLR4, MyD88, p-NF-κB p65, the p-NF-κB p65/NF-κB p65 ratio, and TNF-α/IL-6 levels. Gut microbiota analysis showed increased *Akkermansiaceae* and decreased *Desulfovibrionaceae*. APE also upregulated intestinal Occludin and ZO-1, and downregulated intestinal TNF-α and IL-6. APE prevents NAFLD progression, potentially by regulating gut microbiota, protecting the intestinal mucosal barrier, and inhibiting the LPS/TLR4/MyD88/NF-κB pathway.

## 1. Introduction

Non-alcoholic fatty liver disease (NAFLD) is the most prevalent liver disorder worldwide, with an estimated prevalence of 25–30% among the general adult population [1]. The incidence of NAFLD is substantially higher in males than in females. In the Americas and Southeast Asian countries, the prevalence of NAFLD has already exceeded 40%, and this rate is projected to keep rising over time [2]. In elderly individuals with type 2 diabetes, NAFLD is frequently encountered and tends to heighten the risk of complications such as nonalcoholic steatohepatitis, cirrhosis, and cardiovascular disease [3]. Of note, 10–20% of patients with NAFLD have a normal body mass index and a less severe degree of liver disease, yet they remain at risk of developing steatohepatitis [4]. Thus, arresting the advancement of NAFLD at an early stage is critical for averting or postponing the development of NAFLD-linked hepatitis, cirrhosis, and other disorders.

The pathogenesis of NAFLD remains incompletely understood and is considered multifactorial. The conceptual framework for its development has evolved from the initial “two-hit” hypothesis to the currently more accepted “multiple-hit” paradigm. This latter hypothesis emphasizes interactions between genetic and environmental factors, cross-talk among multiple organs and tissues (including adipose tissue, pancreas, gut, and liver), as well as contributing elements such as insulin resistance, adipokine secretion, nutritional factors, and gut microbiota [5,6]. Despite the continuous evolution of pathogenic theories, no approved pharmacological treatment is yet available for NAFLD or its more severe form, non-alcoholic steatohepatitis (NASH) [7]. In recent years, medicinal plants have garnered significant attention in the exploration of therapeutic approaches for NAFLD [8].

*Anacyclus pyrethrum* (L.) DC, a member of the Asteraceae family [9]. The root of *Anacyclus pyrethrum* is native to Morocco, Algeria, and other regions [9]. Studies have demonstrated that this plant possesses pharmacological properties such as improving glycemic control and providing hepatoprotective effects [10,11,12].

In China, small-scale cultivation of *Anacyclus pyrethrum* root also occurs in the Hetian and Yili regions of Xinjiang, where it is recognized as a distinctive ethnic medicinal plant of the area. In Xinjiang’s traditional medical practice, the dried root is used to treat headache, cold-induced toothache, and neurasthenia. Local hospitals in Xinjiang apply the root of *Anacyclus pyrethrum* for managing pain and cough-variant asthma. Chinese researchers have identified five pairs of alkaloid enantiomers with potent analgesic activity from this Xinjiang ethnic medicine, thereby elucidating the material basis underlying its pain-relieving properties [13]. Additionally, Chinese researchers have discovered that the root of *Anacyclus pyrethrum* can ameliorate Parkinson’s disease with mild cognitive impairment by modulating the gut microbiota and its metabolites [14]. More recently, research has indicated its potential to modulate gut microbiota composition and function [15]. The gut microbiota is inextricably linked to NAFLD and plays a pivotal role in the pathogenesis of NAFLD-related cirrhosis, hepatocellular carcinoma, and other conditions [16].

The human gut harbors approximately 40 trillion microorganisms, with a collective weight of 1–2 kg, functioning as a “hidden organ” that contributes to immunomodulation in metabolic diseases. Within the “multiple-hit” hypothesis framework, gut microbiota plays a significant role in NAFLD progression, particularly through mechanisms involving the gut–liver axis. NAFLD frequently coexists with disruption of the gut microbiota. This disturbance leads to heightened intestinal permeability, which promotes the entry of lipopolysaccharide (LPS) and bacterial translocation through the hepatic portal vein into the liver, thereby accelerating the development and progression of NAFLD [17]. LPS is a surface glycolipid produced by Gram-negative bacteria. It is capable of eliciting both acute and chronic inflammation [18]. Upon disruption of the gut microbiota, the intestinal mucosal barrier becomes compromised; LPS derived from Gram-negative bacteria not only acts locally on the intestine to further damage the mucosal barrier but can also enter the liver via the bloodstream. LPS acts via the TLR4 receptor to induce downstream activation of MyD88/NF-κB p65, thereby eliciting an inflammatory response [19,20]. Gut microbiota dysbiosis disrupts the tight junction protein ZO-1, which raises circulating levels of LPS and activates the TLR4/MyD88/NF-κB p65 pathway, thereby exacerbating the progression of NAFLD [21,22]. The above studies demonstrate that the LPS/TLR4/MyD88/NF-κB p65 axis may represent a key pathway mediating the gut–liver axis to influence the progression of NAFLD.

**Figure 1:**
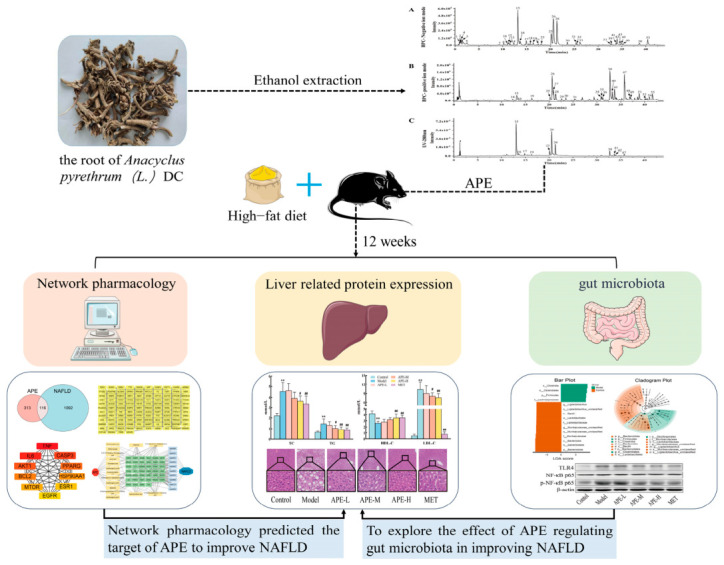
This study used UPLC-Q-TOF/MS to analyze *Anacyclus pyrethrum* extract (APE) components, network pharmacology to predict targets, and a high-fat diet-induced NAFLD mice model to validate APE’s effects on metabolic parameters, hepatic steatosis, and the LPS/TLR4/MyD88/NF-κB pathway, while also analyzing gut microbiota structure and intestinal barrier function, offering a new strategy for NAFLD treatment. ** *p* < 0.01 vs. Control; ^#^ *p* < 0.05, ^##^ *p* < 0.01 vs. Model.

The root of *Anacyclus pyrethrum* demonstrates considerable pharmacological potential, notably exerting anti-inflammatory, anti-diabetic, gut microbiota-modulating, and hepatoprotective actions. However, its effect on improving NAFLD remains unclear. Network pharmacology is an interdisciplinary field that integrates systems biology, bioinformatics, and pharmacology. It can assist researchers in rapidly predicting the signaling pathways through which active ingredients in medicinal plants may improve diseases. The composition of the root of *Anacyclus pyrethrum* is complex. This study plans to analyze its components using UPLC-Q-TOF-MS technology and predict the signaling pathways through which it improves NAFLD. Therefore, this study established a mice NAFLD model using a high-fat diet regimen, intervened with the ethanol extract of *Anacyclus pyrethrum* root (APE), and integrated network pharmacology, in vivo validation, and gut microbiota analysis, proposing the scientific hypothesis that APE may improve NAFLD by mediating the gut–liver axis to regulate the TLR4/MyD88/NF-κB p65 pathway. An overview of the experimental design is depicted in Figure 1.

## 2. Results

### 2.1. Chemical Composition of APE

**Figure 2:**
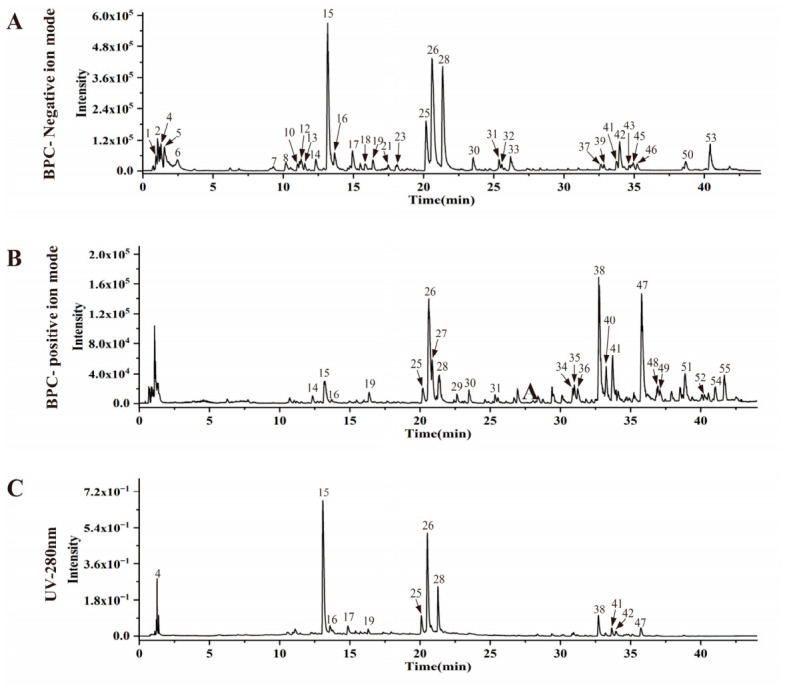
Chromatographic profiles of APE obtained by ultra-performance liquid chromatography coupled with UPLC-Q-TOF/MS. (**A**) UPLC-HRMS BPC for APE acquired in negative ion mode; (**B**) UPLC-HRMS BPC for APE acquired in positive ion mode; (**C**) UV profile of APE monitored at 280 nm.

Chemical characterization of APE utilized UPLC-Q-TOF-MS, with compound identification relying on a high-resolution mass spectrometry database. Fifty-five compounds were successfully identified. Among them were 10 phenolic acids, 10 amides, and 10 fatty acids. Additionally, the extract contained 6 phenols, 4 phenolic acid glycosides, 4 organic acids, 2 alcohol glycosides, 2 flavonoid glycosides, and one compound each of monoterpene glycosides, sugars, and esters (Table 1 and Figure 2).

### 2.2. Investigation of Compound-Target Interactions via Network Pharmacology and Molecular Docking

#### 2.2.1. Common Targets of APE and NAFLD

**Figure 3:**
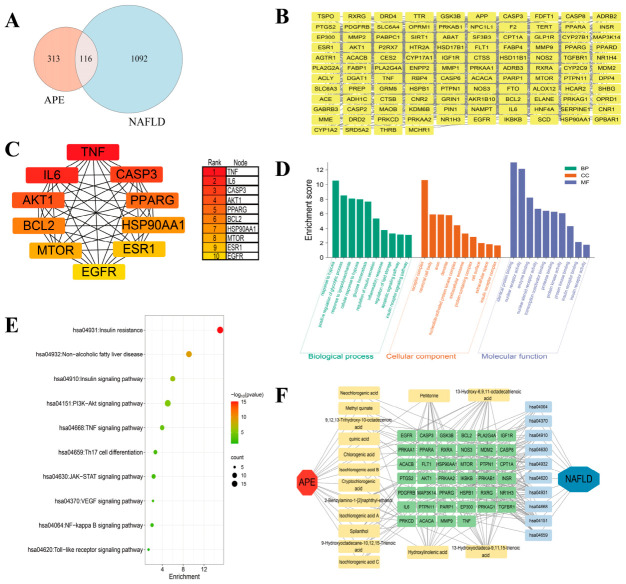
Network pharmacology-based prediction of potential therapeutic targets of APE against NAFLD. (**A**) Venn diagram depicting the shared target genes between APE and NAFLD. (**B**) Protein–protein interaction (PPI) network depicting the core targets, generated using Cytoscape 3.10.2. (**C**) Top 10 core targets of APE against NAFLD, determined based on the PPI network through application of the cytoHubba plugin. (**D**) Gene Ontology (GO) enrichment analysis. (**E**) Kyoto Encyclopedia of Genes and Genomes (KEGG) pathway enrichment analysis. (**F**) Comprehensive “APE-components-targets-pathways-NAFLD” network visualized with Cytoscape 3.10.2.

A total of 20 chemical constituents from APE-including quinic acid, citric acid, isocitric acid, methyl quinate, glucosyringic acid, neochlorogenic acid, dichlorogelignate, chlorogenic acid, cryptochlorogenic acid, isochlorogenic acid B, isochlorogenic acid A, isochlorogenic acid C, 9,12,13-trihydroxy-10-octadecenoic acid, 2-benzylamino-1-[2]naphthyl-ethanol, spilanthol, hydroxylinolenic acid, pellitorine, 13-hydroxyoctadeca-9,11,15-trienoic acid, 9-hydroxyoctadecane-10,12,15-trienoic acid, and 13-hydroxy-6,9,11-octadecatrienoic acid-were analyzed using the Swiss TargetPrediction database. This yielded 1030 targets, which were deduplicated to obtain 429 unique targets. Meanwhile, 1208 NAFLD-related targets were retrieved from the GeneCards database. Intersection analysis identified 116 common targets between APE and NAFLD (Figure 3A,B).

#### 2.2.2. Functional Enrichment Studies of GO and KEGG

The common targets were mapped to the STRING database with a confidence threshold of 0.4, and the resulting network was visualized using Cytoscape 3.10.2. The network comprised 114 nodes and 1126 edges. The cytoHubba plugin was employed to identify the top 10 core targets of APE against NAFLD, including TNF, IL-6, CASP3, AKT1, PPARG, BCL2, HSP90AA1, MTOR, ESR1, and EGFR (Figure 3C).

GO enrichment analysis performed via the DAVID database revealed 410 biological process (BP) terms, primarily associated with response to hypoxia, positive regulation of glycolytic process, response to lipopolysaccharide, cellular response to hypoxia, glucose homeostasis, regulation of insulin secretion, and inflammatory response. For cellular component (CC), 58 terms were identified, mainly involving receptor complex, neuronal cell body, axon, and dendrite. Molecular function (MF) analysis yielded 118 terms. The results encompassed activities related to identical protein binding, notably featured nuclear receptor activity, and extended to enzyme binding and nuclear steroid receptor activity (Figure 3D).

Based on a *p*-value threshold set at <0.05 and potential relevance to NAFLD, 10 pathways were selected from the 132 identified by KEGG pathway analysis. These key pathways included hsa04931 (Insulin resistance), hsa04932 (Non-alcoholic fatty liver disease), hsa04910 (Insulin signaling pathway), hsa04151 (PI3K-Akt signaling pathway), hsa04668 (TNF signaling pathway), hsa04659 (Th17 cell differentiation), hsa04630 (JAK-STAT signaling pathway), hsa04370 (VEGF signaling pathway), hsa04064 (NF-kappa B signaling pathway), and hsa04620 (Toll-like receptor signaling pathway) (Figure 3E). Finally, a comprehensive “APE-components-targets-pathways-NAFLD” network was constructed using Cytoscape 3.10.2 (Figure 3F).

#### 2.2.3. Docking of Key Components with Core Targets

**Figure 4:**
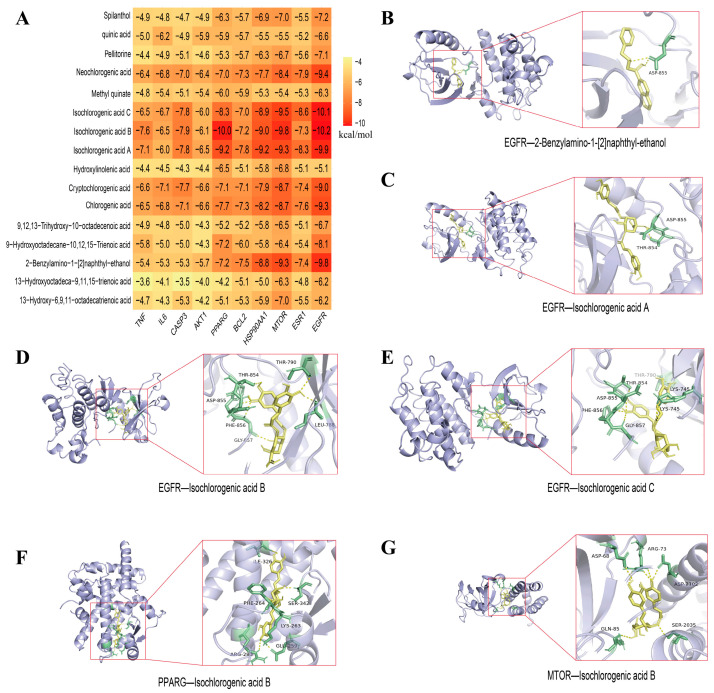
Evaluation of key APE constituents for binding to the top 10 cytoHubba-screened core targets. (**A**) Heatmap illustrating the binding affinities between APE constituents and core targets. (**B**–**E**) Molecular docking models of EGFR with 2-Benzylamino-1-[2]naphthyl-ethanol, Isochlorogenic acids A, B, and C, respectively. (**F**) Docking model of PPARG with Isochlorogenic acid B. (**G**) Docking model of MTOR with Isochlorogenic acid B.

This analysis involved molecular docking between the key bioactive components of APE (Figure 3F) and the top 10 core targets prioritized by cytoHubba. The results revealed strong binding affinity (binding energy ≤ −9.8 kcal/mol) between EGFR and four compounds: 2-Benzylamino-1-[2]naphthyl-ethanol, and Isochlorogenic acids A, B, and C. Additionally, PPARG showed high affinity with Isochlorogenic acid B, and MTOR demonstrated strong binding with Isochlorogenic acid B. These findings suggest stable binding interactions between these compounds and their respective targets (Figure 4).

### 2.3. Therapeutic Effects of APE on HFD-Induced NAFLD and Glucose-Lipid Metabolic Disorders

#### 2.3.1. Body Weight

**Figure 5:**
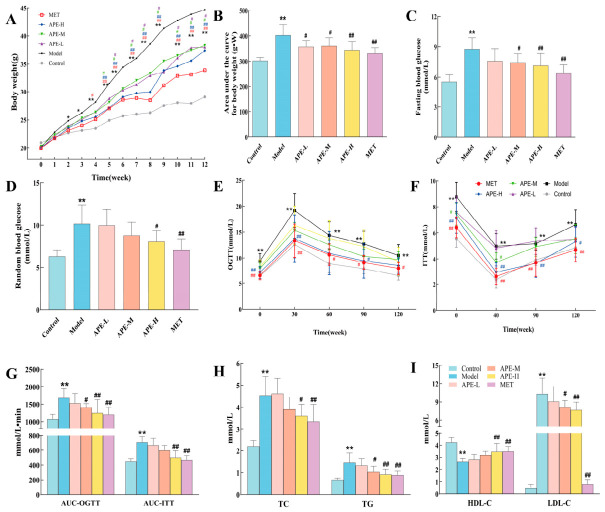
Effects of APE on body weight, blood glucose, and serum lipid levels in HFD-fed mice. (**A**) Body weight changes over 0–12 weeks. (**B**) Area under the body weight curve during 0–12 weeks. (**C**,**D**) Fasting and random blood glucose profiles in week 11. (**E**) OGTT curves in week 11. (**F**) ITT curves in week 11. (**G**) AUC for OGTT and ITT in week 11. (**H**) Serum TC and TG levels at week 12. (**I**) Serum HDL-C and LDL-C levels at week 12. Data are displayed as mean ± SD. Statistical significances are denoted as follows: * *p* < 0.05 and ** *p* < 0.01 versus the Control group; ^#^ *p* < 0.05 and ^##^ *p* < 0.01 versus the Model group. In (**A**,**E**,**F**), the different colors of ^#^ or ^##^: purple, green, blue, and red represent the differences of APE-L, APE-M, APE-H, and MET compared to the Model, respectively.

Using repeated-measures ANOVA, the results showed that Mauchly’s Test had a *p*-value < 0.05, and Greenhouse–Geisser correction was applied. Confidence interval adjustment was performed using the Bonferroni method. The within-subjects effects (time; time × group interaction) and the between-subjects effect (group) were all significant at *p* < 0.001. The time effect from the within-subjects effect test indicated that mouse body weight increased significantly over time, with a statistically significant difference (*F* = 591.445, *p* < 0.001). The time × group interaction suggests that body weight changes over time differed significantly among groups (*F* = 12.856, *p* < 0.001). The between-subjects effect test, followed by post hoc testing using the Bonferroni correction, revealed significant differences in body weight among the groups (*F* = 13.189, *p* < 0.001) (Table 2). The line graph of body weight changes in mice from weeks 0 to 12 and the analysis of the total body weight curve (AUC 0–12 weeks) are presented in Figure 5A,B.

#### 2.3.2. Blood Glucose and Lipid Profiles

At week 11, glucose metabolism was evaluated. Mice in the Model group exhibited significantly higher areas under the curve for both OGTT and ITT (AUC-OGTT and AUC-ITT) than those in the Control group (*p* < 0.05). When compared with the Model group, the APE-H, and MET treatments showed a significant reduction in both AUC-OGTT and AUC-ITT (*p* < 0.05) (Figure 5C–G).

At week 12, serum lipid levels were measured. The Control group values were used as a baseline, revealing that the Model group developed a distinct dyslipidemic profile, with significant elevations in TC, TG, and LDL-C, coupled with a reduction in HDL-C (*p* < 0.05). APE-H and MET treatment significantly reversed these changes with a reduction in TC, TG, and LDL-C and an increase in HDL-C compared to the Model group (*p* < 0.05) (Figure 5H–I).

#### 2.3.3. Liver Histopathology

**Figure 6:**
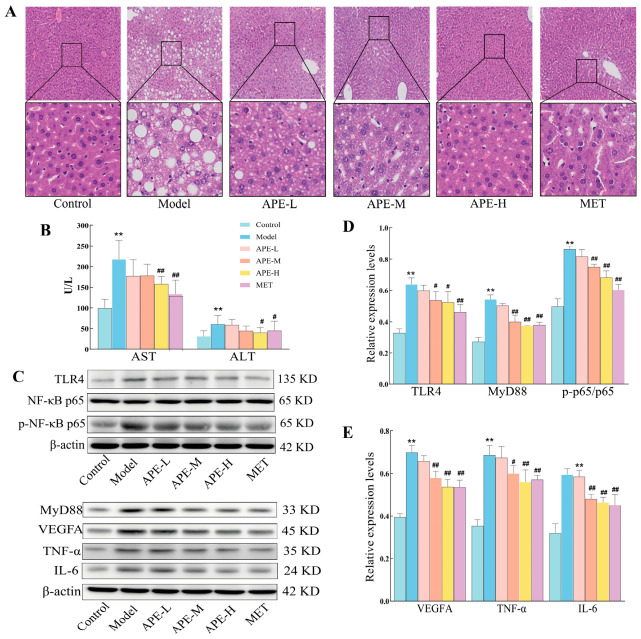
Role of APE in regulating hepatic histopathology and protein expression in a mice model of HFD-induced liver injury. (**A**) Illustrative micrographs of H&E-stained liver tissues (100 μm scale). (**B**) Circulating levels of the hepatotoxicity indicators AST and ALT. (**C**) Protein expression in liver tissues detected by Western blot. (**D**,**E**) Quantitative analysis of protein expression levels. mean ± SD. ** *p* < 0.01 vs. Control; ^#^ *p* < 0.05, ^##^ *p* < 0.01 vs. Model.

H&E staining was applied for the assessment of hepatic histopathological changes following high-fat diet administration. In the Control group, hepatocytes were uniform in size and tightly arranged. A distinct contrast was observed in the Model group, which developed a disordered hepatic cord architecture and marked hepatocellular steatosis, featuring cytoplasmic vacuolization and pale staining. The pathological features observed in the Model group, including vacuolar degeneration and disrupted hepatocyte arrangement, were markedly ameliorated by treatment with either APE or MET. The protective effect was dose-dependent, with the APE-H group demonstrating hepatic morphology nearly resembling that of the Control group (Figure 6A).

#### 2.3.4. Liver Function and Hepatic Protein Expression

A statistically significant rise (*p* < 0.05) in serum AST and ALT levels was observed in the Model group relative to the Control group. A significant reduction (*p* < 0.05) in these levels was observed in the APE-H and MET groups when set against the Model group (Figure 6B).

Levels of TLR4, MyD88, p-NF-κB p65 (and its ratio to total NF-κB p65), VEGF, TNF-α, and IL-6 were significantly more elevated in the Model group than in controls (*p* < 0.05), as determined by Western blot. This effect was antagonized by intervention with APE-M, APE-H, or MET, which all produced a significant downregulation of these markers—namely, TLR4, MyD88, p-NF-κB p65 (and its ratio to total NF-κB p65), VEGF, TNF-α, and IL-6—compared to the Model group (*p* < 0.05, Figure 6C–E).

### 2.4. Effects of APE on Gut Microbiota and Intestinal Mucosal Barrier

#### 2.4.1. Colonic Histopathology in Mice

**Figure 7:**
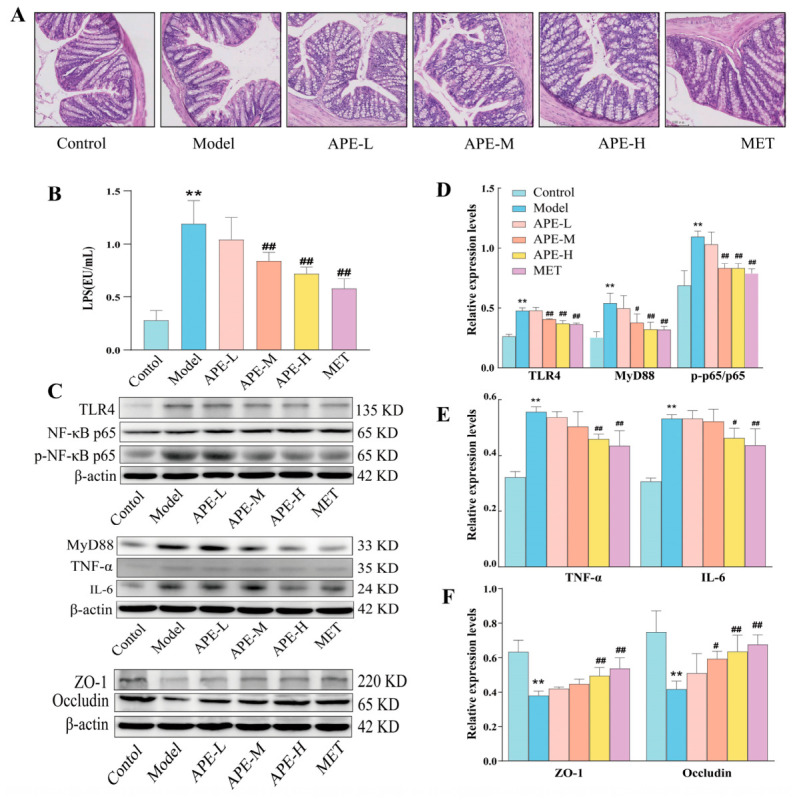
Effects of APE on colonic histopathology and related protein expression in HFD-fed mice. (**A**) Depicts representative H&E-stained colon histology (100 μm scale). (**B**) Presents the measured serum LPS levels for each group. (**C**–**F**) Protein expression in colon tissues. Data represent mean ± SD. Symbols denote: ** *p* < 0.01 vs. Control; ^#^ *p* < 0.05, ^##^ *p* < 0.01 vs. Model.

The Control group demonstrated normal colonic histology in H&E-stained sections, featuring an intact and continuous mucosal epithelium, clear cellular stratification, a high abundance of goblet cells, and well-maintained glands in the lamina propria. In contrast to the well-preserved Control morphology, the Model group exhibited disrupted mucosal integrity and a marked reduction in goblet cells, a pathology that was significantly ameliorated by all APE treatments and MET (Figure 7A).

#### 2.4.2. Intestinal Barrier Proteins and Serum LPS Levels

A significant elevation in serum LPS was observed in the Model group compared to the Control group. Conversely, intervention with APE-M, APE-H, or MET effectively suppressed circulating LPS levels (Figure 7B).

Western blot analysis revealed a pronounced inflammatory and barrier dysfunction profile in the Model group, characterized by upregulation of proteins including TLR4, MyD88, p-NF-κB p65 (alongside its ratio to total NF-κB p65), TNF-α, and IL-6, concurrent with downregulation of ZO-1 and Occludin (*p* < 0.05). Treatment with APE-M, APE-H, or MET significantly restored this imbalance, suppressing the pro-inflammatory signaling axis by notably reducing TLR4, MyD88, p-NF-κB p65 (and its p-p65/t-p65 ratio), TNF-α, and IL-6 and upregulating Occludin. Furthermore, APE-H and MET specifically enhanced ZO-1 expression (*p* < 0.05 for all comparisons; Figure 7C–F).

#### 2.4.3. Impact of APE on Gut Microbiota Composition in Mice

**Figure 8:**
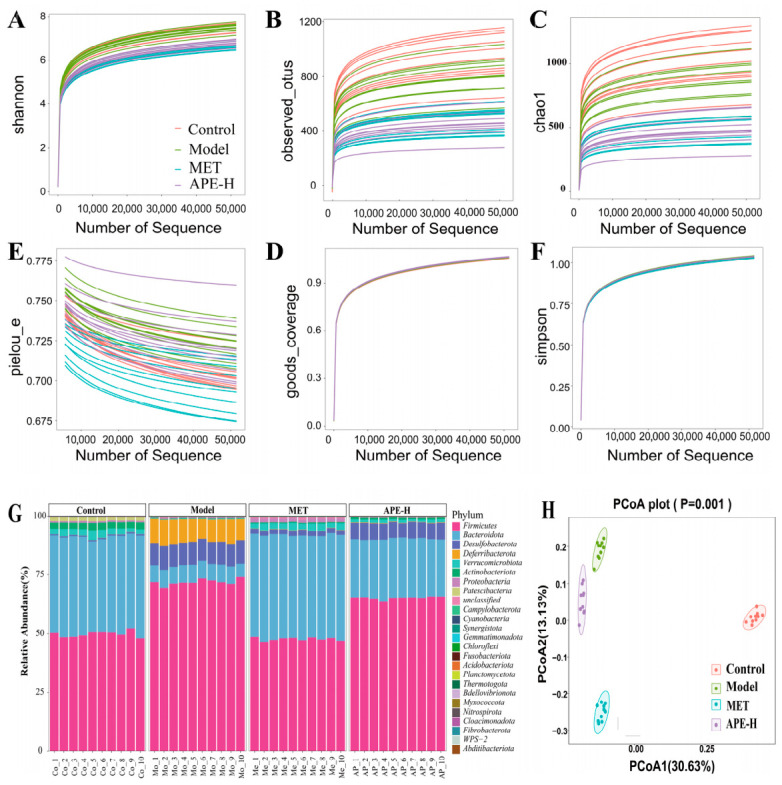
Gut microbiota composition in Control, Model, high-dose APE (APE-H), and metformin (MET) groups. (**A**–**F**) These are the α-diversity analysis plots of gut microbiota: Shannon, observed_otus, Chao1, pielou_e, goods_coverage, and Simpson, respectively. (**G**) Stacked bar chart of phylum-level bacterial abundance in the gut of each mice in each group. (**H**) PCoA plot of gut microbiota from four groups of mice based on the unweighted UniFrac algorithm.

Sequencing of gut microbiota was performed on four groups (Control, Model, APE-H, and MET). Alpha diversity analyses including Shannon, observed_otus, Chao1, pielou_e, goods_coverage, and Simpson showed that the samples had rich bacterial species, good completeness, and high microbial diversity (Figure 8A–F). Meanwhile, the relative abundance of gut bacteria at the phylum level in each group was analyzed (Figure 8G). The PCoA plot based on the unweighted UniFrac algorithm showed that the microbial communities of the four groups were relatively scattered, indicating differences in microbiota structure (Figure 8H).

**Figure 9:**
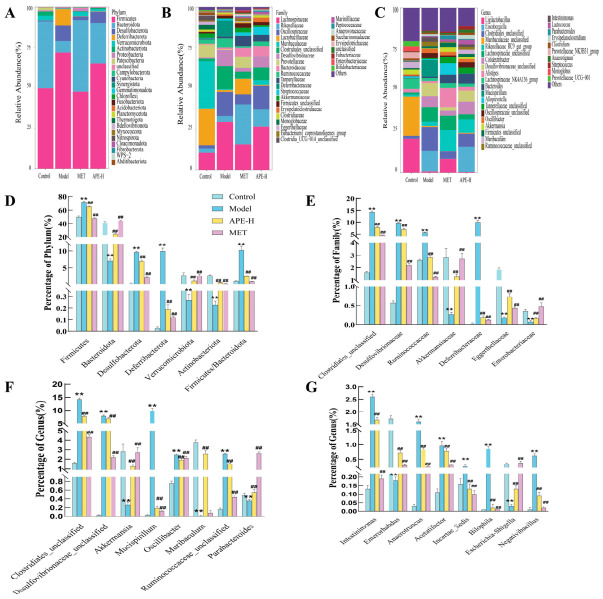
Gut microbiota composition in Control, Model, high-dose APE (APE-H), and metformin (MET) groups. (**A**–**C**) Stacked bar charts showing the relative abundances of gut bacteria at the phylum, family, and genus levels, respectively, at week 12. (**D**,**E**) Bar graphs illustrating differentially abundant bacterial phyla and families among groups at week 12. (**F**,**G**) Bar graphs showing differentially abundant bacterial genera among groups at week 12. All values mean ± SD (** *p* < 0.01 vs. Control; ^##^ *p* < 0.01 vs. Model).

The dominant bacterial phyla across all four groups comprised *Firmicutes*, *Bacteroidota*, *Desulfobacterota*, *Deferribacterota*, *Verrucomicrobiota*, and *Actinobacteriota*. A distinct shift in the microbial composition was observed in the Model group relative to the Control group (*p* < 0.05). This shift was characterized by a marked increase in the representation of *Firmicutes*, *Desulfobacterota*, and *Deferribacterota*, as well as the *Firmicutes/Bacteroidota* ratio, contrasting with a significant reduction in *Bacteroidota*, *Verrucomicrobiota*, and *Actinobacteriota*. The Model group-induced microbial alterations were significantly mitigated following treatment with either APE-H or MET. Specifically, these treatments lowered the abundances of *Firmicutes*, *Desulfobacterota*, and *Deferribacterota* and the *Firmicutes/Bacteroidota* ratio, while promoting the growth of *Bacteroidota*, *Verrucomicrobiota*, and *Actinobacteriota* (*p* < 0.05) (Figure 9A,D).

Microbial community profiles at the family level significantly distinguished the Model from the Control groups, with the former showing increases in *Clostridiales_unclassified*, *Desulfovibrionaceae*, *Ruminococcaceae*, and *Deferribacteraceae* and decreases in *Akkermansiaceae*, *Eggerthellaceae*, and *Enterobacteriaceae* (all *p* < 0.05). Intervention with APE-H or MET largely reversed this profile, reducing the model-elevated families and promoting the growth of those that were suppressed (all *p* < 0.05) (Figure 9B,E).

The Model group exhibited a significant shift in genus-level abundances, including increases in *Clostridiales_unclassified*, *Desulfovibrionaceae_unclassified*, *Mucispirillum*, *Oscillibacter*, *Ruminococcaceae_unclassified*, *Intestinimonas*, *Anaerotruncus*, *Acetatifactor*, *Incertae_Sedis*, *Bilophila*, and *Negativibacillus* and decreases in *Akkermansia*, *Muribaculum*, *Parabacteroides*, *Enterorhabdus*, *and Escherichia-Shigella* relative to controls (*p* < 0.05). These shifts were effectively mitigated by both APE-H and MET treatment, which significantly modulated the affected genera back toward normal levels (*p* < 0.05) (Figure 9C,F,G).

**Figure 10:**
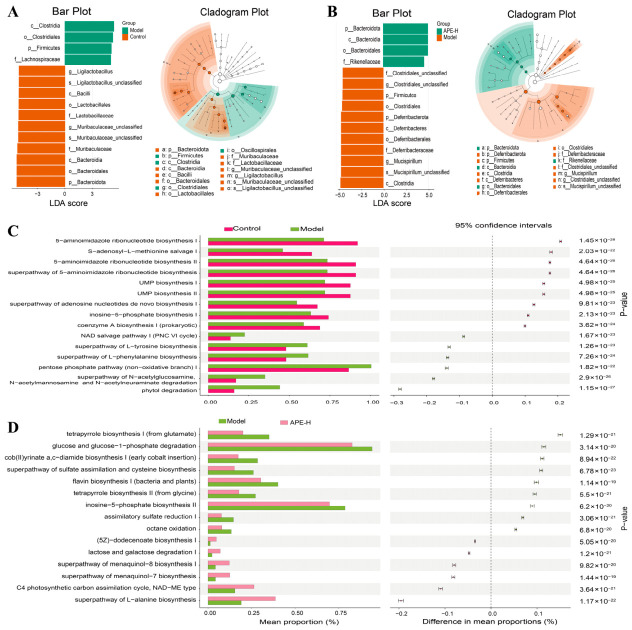
Association between the core gut microbiota and predicted metabolic outputs in Control, Model, and high-dose APE (APE-H) treated mice. (**A**,**B**) This analysis presents the LDA scores (LDA score > 3) for the top 15 significantly different gut microbial taxa across two key comparisons: Control versus Model groups, and Model versus APE-H groups, supplemented by LEfSe-generated cladograms that illustrate the taxonomic relationships. (**C**,**D**) Predicted metabolic phenotypes of gut microbiota for the Control vs. Model groups and the Model vs. APE-H groups, respectively.

To identify signature microbial taxa, LEfSe analysis was performed using logarithmic LDA scores and cladogram visualization. This revealed the top 15 differentially abundant taxa across the following comparisons: Control group against Model group, and Model group against APE-H group (Figure 10A,B). Given these structural alterations, we further employed PICRUSt for functional prediction and integrated the CAZy database (focusing on carbohydrate-active enzymes) to explore metabolic characteristics of the gut microbiota. The top 15 significantly altered metabolic functions based on Welch’s *t*-test (*p* < 0.05) are presented. Compared with the Control group, the Model group showed downregulation in the following pathways: 5-aminoimidazole ribonucleotide biosynthesis I, S-adenosyl-L-methionine salvage I, 5-aminoimidazole ribonucleotide biosynthesis II, superpathway of 5-aminoimidazole ribonucleotide biosynthesis, UMP biosynthesis I, UMP biosynthesis II, superpathway of adenosine nucleotides de novo biosynthesis I, inosine-5-phosphate biosynthesis I, and coenzyme A biosynthesis I (prokaryotic). Conversely, the following pathways were upregulated: NAD salvage pathway I (PNC VI cycle), superpathway of L-tyrosine biosynthesis, superpathway of L-phenylalanine biosynthesis, pentose phosphate pathway (non-oxidative branch) I, superpathway of N-acetylglucosamine, N-acetylmannosamine and N-acetylneuraminate degradation, and phytol degradation.

Relative to the Model group, APE-H treatment resulted in significant downregulation in tetrapyrrole biosynthesis I (from glutamate), glucose and glucose-1-phosphate degradation, cob(II)yrinate a,c-diamide biosynthesis I (early cobalt insertion), superpathway of sulfate assimilation and cysteine biosynthesis, flavin biosynthesis I (bacteria and plants), tetrapyrrole biosynthesis II (from glycine), inosine-5-phosphate biosynthesis II, assimilatory sulfate reduction I, and octane oxidation. Meanwhile, the following pathways were markedly elevated: (5Z)-dodecenoate biosynthesis I, lactose and galactose degradation I, superpathway of menaquinol-8 biosynthesis I, superpathway of menaquinol-7 biosynthesis, C4 photosynthetic carbon assimilation cycle (NAD-ME type), and superpathway of L-alanine biosynthesis (Figure 10C,D).

### 2.5. Correlation Analysis

**Figure 11:**
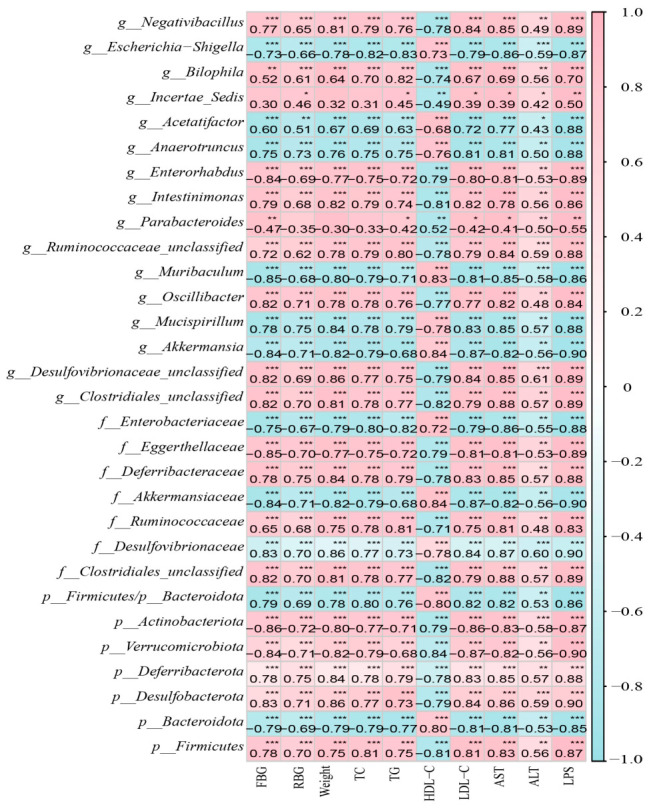
Correlation analysis between intestinal microbial abundance and levels of fasting blood glucose (FBG), random blood glucose (RBG), weight, total cholesterol (TC), triglycerides (TG), HDL-C, LDL-C, AST, ALT, and lipopolysaccharide (LPS) in the control, model, and APE-H groups. The values in the figure represent Spearman’s correlation coefficients. Symbols denote: * *p* < 0.05, ** *p* < 0.01, *** *p* < 0.001.

To evaluate associations between gut microbiota and host physiology, a Spearman correlation analysis was conducted using a suite of metabolic parameters (comprehensive lipid profiles, liver function markers, glycemia, body weight, and serum LPS) and microbial abundance data derived from the Control, Model, and APE-H groups (Figure 11). *Akkermansiaceae* showed significant negative correlations with fasting blood glucose, TC, TG, AST, ALT, and serum LPS levels, whereas *Desulfovibrionaceae* exhibited significant positive correlations with these parameters (*p* < 0.01).

## 3. Discussion

A NAFLD exists within a spectrum of metabolic disorders that includes insulin resistance, type 2 diabetes, hyperlipidemia, and obesity. Although the precise etiology of NAFLD remains incompletely understood, high-fat diet is strongly implicated in the pathogenesis of NASH, promoting both initial hepatic steatosis and disease progression [23]. Currently, there is a lack of clinically approved drugs for the prevention and treatment of NAFLD. Medicinal plants and their active constituents have thus become an important source for exploring potential NAFLD therapies. For instance, Shang et al. reported that the Shenge Formula conferred hepatoprotection and alleviated insulin resistance in mice following NAFLD elicited by a high-fat diet, following an 8-week intervention with metformin as a positive control [24]. According to Zheng et al., Astragalus polysaccharides mitigated NAFLD in mice with diet-induced obesity by remodeling systemic and hepatic bile acid profiles [25]. By mediating anti-inflammatory effects and gut microbiota composition, the traditional Chinese formula Sinisan effectively mitigates NAFLD in mice fed a high-fat diet [26]. In addition, conifer polysaccharides were found to prevent NAFLD by regulating lipogenesis, reducing inflammation and oxidative stress, and enhancing mitochondrial function [27]. These studies collectively suggest that medicinal plants, either as single agents or in formulated preparations, hold promise in the prevention and treatment of NAFLD.

As a medicinal plant, both the aqueous and ethanol extracts of *Anacyclus pyrethrum* root have demonstrated anti-diabetic effects by reducing blood glucose in streptozotocin-induced diabetic rats and significantly inhibiting α-amylase activity [10,11]. Furthermore, Usmani et al. reported that *Anacyclus pyrethrum* root ameliorates drug-induced liver injury caused by isoniazid and rifampicin combination, not only by lowering liver enzyme levels but also by improving liver histopathology, with efficacy comparable to the hepatoprotective agent silymarin [12]. These findings suggest that *Anacyclus pyrethrum* root possesses both glucose-regulating and hepatoprotective properties. Based on this evidence, the present study aimed to evaluate the effect of a 12-week intervention with APE in mice with high-fat diet-induced NAFLD. Our findings indicate that APE consistently improved glycemic control across multiple assays, counteracting the HFD-induced elevations in fasting blood glucose, random blood glucose, and impairments in both oral glucose and insulin tolerance tests. The treatment also demonstrated efficacy in reducing key serum parameters, including triglyceride and total cholesterol levels, as well as the activities of aspartate aminotransferase and alanine aminotransferase. These findings indicate that APE can partially attenuate HFD-induced insulin resistance and exert hepatoprotective effects, thereby slowing the progression of NAFLD. This supports the important role of insulin resistance within the “multiple-hit” hypothesis of NAFLD pathogenesis.

Network pharmacology systematically analyzes the complex network relationships among drugs, targets, and diseases, revealing the synergistic mechanisms of multi-target drug actions, accelerating drug discovery and repositioning, and offering theoretical support for advancing traditional Chinese medicine. Currently, identifying the therapeutic targets of medicinal plants for disease amelioration remains a critical challenge to be addressed.

This research employed network pharmacology to elucidate the mechanisms by which APE’s active constituents alleviate NAFLD. First, UPLC-Q-TOF-MS analysis revealed that phenolic acids, amides, and fatty acids are the major constituents of APE. Compounds including chlorogenic acid, neochlorogenic acid, and isochlorogenic acids A and B, along with spilanthol were identified as key components. Chlorogenic acid has been found to directly bind to myeloid differentiation primary response 88 (MyD88), competitively inhibiting the interaction between Toll-like receptor 4 (TLR4) and MyD88, thereby mitigating LPS-TLR4-MyD88-induced hepatic inflammation and attenuating the progression of NASH [28]. Additionally, chlorogenic acid reduces plasma total cholesterol and LDL levels induced by a high-cholesterol diet and decreases hepatic lipid accumulation, exerting hepatoprotective effects [29]. Neochlorogenic acid significantly ameliorates high-fat diet-induced disturbances in serum lipid profiles, hepatic lipid deposition, and reduced hepatic antioxidant activity [30]. These isomers, isochlorogenic acid A and B, suppress the expression of key lipogenic regulators—namely fatty acid synthase (FAS), acetyl-CoA carboxylase (ACC), and peroxisome proliferator-activated receptor γ (PPARγ)—indicating their potential as effective agents for NAFLD treatment [31]. PPARγ, a ligand-activated transcription factor belonging to the nuclear hormone receptor superfamily, is considered a key metabolic regulator in hepatic lipid metabolism and inflammation, participating in NAFLD pathogenesis through lipogenesis, insulin resistance, inflammation, oxidative stress, endoplasmic reticulum stress, and fibrosis [32]. Spilanthol also reduces the expression of FAS and ACC, decreasing body weight and visceral adipose tissue weight in high-fat diet-fed mice [33]. FASN is a key enzyme catalyzing the final step of fatty acid biosynthesis, determining the capacity for fatty acid synthesis. Elevated FASN levels are observed in both NASH patients and mice models, making it a significant biomarker for NAFLD diagnosis and therapeutic development [34]. De novo lipogenesis (DNL) is a critical factor driving hepatic steatosis and is associated with hepatic inflammation and fibrosis. Thus, inhibiting its rate-limiting enzyme, acetyl-CoA carboxylase, represents a promising therapeutic strategy for NAFLD [35]. Multiple compounds—chlorogenic acid, its analogs (neochlorogenic acid, isochlorogenic acids A/B), and spilanthol—are collectively evidenced to exert lipid-lowering and hepatoprotective effects. Docking studies confirmed high-affinity binding (≤−6.0 kcal/mol) between PPARG (PPARγ) and the active constituents—isochlorogenic acids A–C, chlorogenic acid, and spilanthol. As constituents of APE, these compounds indicate that APE may exert lipid-lowering, anti-inflammatory, and NAFLD-ameliorating effects by multi-target inhibition of ACC, FAS, and PPARγ expression.

KEGG pathway prediction based on the core components of APE revealed that key mechanisms for APE’s amelioration of NAFLD likely involve several pathways, notably the Insulin signaling pathway, Insulin resistance, NAFLD, TNF signaling pathway, NF-kappa B signaling pathway, and Toll-like receptor signaling pathway. Further investigation into the liver tissue of NAFLD mice via Western blot revealed that APE treatment effectively curtailed activation in the TLR4/MyD88/NF-κB pathway, which translated into lower levels of the pro-inflammatory proteins TNF-α and IL-6. Zhang et al. fed TLR4 knockout mice a high-fat diet and subjected them to ischemia–reperfusion treatment, and found that the expression of IL-6 and TNF-α was significantly reduced [36]. Studies suggest that steatosis, oxidative stress, and the presence of inflammatory mediators such as TNF-α and IL-6 are associated with alterations in nuclear factors in NAFLD [37]. Elevated concentrations of IL-6 and TNF-α in the blood are significantly associated with an increased risk of NAFLD and may serve as biomarkers for patients with NAFLD [38]. Consistent with the aforementioned studies, IL-6 and TNF-α are downstream inflammatory molecules of the TLR4 pathway and key factors influencing the progression of NAFLD. Through in vivo animal experiments, the present study confirmed that APE inhibits the TLR4 pathway and reduces the hepatic expression levels of IL-6 and TNF-α, thereby validating the network pharmacology–based prediction that APE mediates the TLR4 pathway to ameliorate NAFLD. Moreover, network pharmacology in this study identified IL-6 and TNF-α as important targets of APE in improving NAFLD, suggesting that APE can modulate the expression of these two cytokines. Collectively, our findings indicate that APE may lower inflammatory levels via two mechanisms—namely, suppression of the TLR4 pathway and direct action on IL-6 and TNF-α targets—thus exerting multi-target effects to improve NAFLD.

According to Xue et al., daily 2-h exposure to 4% H_2_ in a model of diet-induced NAFLD established in rats reduced hepatic inflammatory gene expression, modulated the gut microbiome, and likely mitigated NAFLD by targeting the LPS/TLR4/NF-κB signaling cascade [39]. Similarly, dihydromyricetin was shown to modulate gut microbiota, reduce serum levels of LPS, IL-1β, and TNF-α, and inhibit the expression of hepatic TLR4 and NF-κB p65, thereby effectively attenuating the progression of NAFLD [40]. Consistent with these findings, our study observed that a high-fat diet increased serum LPS levels in mice, while both APE and MET treatments significantly reduced LPS levels. Serum endotoxin LPS is primarily derived from gut microbiota. Upon crossing the intestinal barrier, LPS is transported via the portal vein to the liver, where it activates the TLR4/NF-κB signaling pathway and exacerbates the progression of NAFLD.

Gut microbiota represents one of the key factors influencing NAFLD progression within the “multiple-hit” hypothesis. A high-fat diet induces structural imbalance in the gut microbiota, triggers inflammation, impairs the intestinal mucosal barrier, compromises epithelial integrity, and increases intestinal permeability in NAFLD mice [41]. Specifically, the *Firmicutes*/*Bacteroidota* ratio is promoted by a high-fat diet and is associated with enhanced inflammatory responses [42]. *Akkermansia muciniphila*, a species belonging to the *Verrucomicrobiota* phylum, primarily colonizes the intestinal mucus layer. It modulates the production of key tight junction proteins like occludin through extracellular vesicles, a mechanism that strengthens intestinal barrier function. Additionally, it produces short-chain fatty acids that suppress the NF-κB pathway, exerting anti-inflammatory effects [43]. *Akkermansia muciniphila* also promotes the transport of L-aspartate from the gut to the liver, activating the LKB1-AMPK axis and stimulating lipid oxidation, which contributes to the amelioration of metabolic-associated fatty liver disease [44]. Metformin has been shown to boost the relative representation of *Akkermansia muciniphila* resident in the mice gut, thereby improving NAFLD [45]. Furthermore, *Akkermansia muciniphila* alleviates high-fat diet-induced obesity, hepatic steatosis, liver injury, and improves glucose tolerance and intestinal barrier function in rats [46]. As such, *Akkermansia muciniphila* is considered a next-generation probiotic with potential for NAFLD intervention [47]. The relative abundance of *Parabacteroides* is negatively correlated with TNF-α mRNA expression and hepatic TG levels, and its reduction may be associated with an increased risk of NAFLD [48]. Dietary fiber from *Dendrobium officinale* can prevent obesity from HFD and improve glucose metabolism through elevating the relative levels of *Muribaculum* in the mice gut [49]. Conversely, a significant decrease in *Muribaculum* abundance may contribute to inflammatory conditions [50]. *Desulfovibrio*, a genus within the class *Deltaproteobacteria*, accounts for approximately 66% of sulfate-reducing bacteria in the human colon. It utilizes sulfate as a terminal electron acceptor to produce hydrogen sulfide (H_2_S) during ATP generation [51]. H_2_S can inhibit mitochondrial respiration in colonic epithelial cells [52]. High-fat diets elevate LPS levels in fecal and serum samples, which may be linked to an increase in LPS-producing bacteria such as *Desulfovibrio* and impaired intestinal integrity [53]. Similarly, *Mucispirillum*, which also inhabits the intestinal mucus layer, is associated with pro-inflammatory responses in the mucosa [54]. *Oscillibacter* has been closely linked to obesity and impaired intestinal permeability [55].

Functional analysis of the gut microbiota in NAFLD mice based on PICRUSt showed that APE significantly enhanced the superpathway of menaquinol-8 biosynthesis I and the superpathway of menaquinol-7 biosynthesis. Menaquinone, also known as vitamin K2, is a fat-soluble vitamin that can be synthesized in the human gut by both Gram-positive and Gram-negative bacteria [56]. Zhao et al. found that vitamin K2 can reduce body weight, waist circumference, and body fat percentage in mice fed a high-fat diet, and alleviate hepatic steatosis [57]. These findings suggest that APE may promote vitamin K2 synthesis by modulating the gut microbiota, thereby ameliorating NAFLD. Moreover, APE significantly attenuates the superpathway of sulfate assimilation and cysteine biosynthesis and the assimilatory sulfate reduction I pathway. The assimilatory sulfate reduction pathway is an endogenous H_2_S production pathway [58]. In the gut, two major microbial groups--cysteine fermenters and sulfate-reducing bacteria—produce large amounts of hydrogen sulfide. The former include *Escherichia coli*, *Salmonella enterica*, *Clostridia*, and *Enterobacter*, while the latter include *Desulfovibrio*, *Desulfobacter*, *Desulfobulbus*, and *Desulfotomaculum*. Cysteine fermenters generate hydrogen sulfide by fermenting cysteine via cysteine desulfhydrase, whereas sulfate-reducing bacteria produce H_2_S either by reducing inorganic sulfate or through microbial catabolism of sulfated mucins [59]. The results of this study suggest that APE ameliorates NAFLD by reducing H_2_S production and promoting vitamin K2 synthesis.

Therefore, a synthesis of the above literature suggests that a high-fat diet can induce gut microbiota dysbiosis in mice, promoting the increase in harmful bacteria such as *Desulfovibrio*, *Mucispirillum*, *and Oscillibacter*, while inhibiting the growth in beneficial bacterium *Akkermansia muciniphila. Desulfovibrio* produces large amounts of H_2_S, which disrupts the morphology and function of intestinal epithelial cells, promotes the destruction of the intestinal mucosal barrier, and reduces the expression levels of ZO-1 and Occludin proteins. Additionally, *Desulfovibrio* produces LPS. Following disruption of the intestinal mucosa, LPS enters the circulatory system, activates the TLR4/NF-κB pathway, and increases the expression of inflammatory cytokines such as IL-6 and TNF-α, ultimately exacerbating the progression of NAFLD. LPS is a TLR4 ligand; its translocation from the intestinal lumen into the bloodstream activates immune cells in the liver, thereby triggering inflammation, whereas homozygous TLR4-deficient mice are protected from diet-induced fatty liver and hepatic inflammation [60]. *Desulfovibrio desulfuricans* exacerbates atherosclerosis in Apoe^−/−^ mice by increasing intestinal permeability, promoting the entry of LPS into the bloodstream, and activating the TLR4/NF-κB pathway; whereas the TLR4 inhibitor TAK-242 blocks this pathway and alleviates disease progression [61]. *Akkermansia muciniphila* protects the intestinal mucosal barrier, reduces LPS translocation, thereby inhibiting the gut–liver–axis–mediated TLR4/NF-κB pathway and alleviating inflammatory responses.

In this study, APE lowered the intestinal abundance of *Desulfovibrio* and the serum level of LPS in high-fat diet-induced NAFLD mice, while simultaneously enhancing the expression of intestinal mucosal proteins ZO-1 and Occludin, decreasing ALT and AST levels, and ameliorating liver histopathological changes. Notably, PICRUSt analysis suggested that APE may act by diminishing *Desulfovibrio* abundance in the mice gut and, through the superpathway of sulfate assimilation and cysteine biosynthesis as well as the assimilatory sulfate reduction I pathway, lowering H_2_S production. This alleviates disruption of the intestinal mucosal barrier, curtails the translocation of LPS from the gut into the circulatory system, and thereby suppresses the hepatic TLR4/NF-κB pathway, attenuating liver inflammation and exerting a beneficial effect on NAFLD. Furthermore, the reduced abundance of *Desulfovibrio* also led to diminished gut-derived LPS production, whereas the elevated abundance of *Akkermansia muciniphila* strengthened intestinal mucosal barrier function and lessened circulating LPS levels, further contributing to NAFLD improvement. These findings elucidate the mechanism by which APE modulates the gut–liver axis, curtailing LPS production and restraining the TLR4/NF-κB pathway to ameliorate NAFLD.

Nevertheless, this study has certain limitations. While we have provided preliminary evidence from a gut microbiota perspective that APE may suppress inflammation through modulation of the gut microbiota, APE also markedly reduces body weight in high-fat diet-induced NAFLD mice. It remains unclear whether this reduction is an indirect consequence of decreased body fat. Hence, future studies should further examine the food intake of the mice. Other limitations also exist. For instance, we did not utilize TLR4 knockout mice or the TLR4 inhibitor TAK-242 to validate the mechanism by which APE suppresses the TLR4/NF-κB pathway via the gut–liver axis in ameliorating NAFLD. Active compound isolation was not performed to identify important monomeric components responsible for the beneficial effects of APE on NAFLD. Moreover, proteomic or transcriptomic analyses were not conducted to investigate additional targets of APE in NAFLD treatment. Future studies will address these aspects to fully elaborate the multi-target action of APE in ameliorating NAFLD.

## 4. Materials and Methods

### 4.1. Experimental Drugs

The root of *Anacyclus pyrethrum* was sourced from Xinjiang Bencaotang Pharmaceutical Co., Ltd. (Xinjiang, China) (Urumqi Economic and Technological Development Zone, China. Batch No.: 704025).

### 4.2. Extraction and Chemical Analysis of APE

The extraction of APE was performed by refluxing the root material with 65% ethanol at a solid-to-solvent ratio of 1:6 (g/mL) lasting 2 h at 50 °C. The resulting concentration of the extract was carried out using a rotary evaporator and then lyophilized into a dry powder for storage at 4 °C until subsequent use.

For chemical characterization, the test sample was prepared by transferring 2 mL of the extract solution into a centrifuge tube. Prior to analysis, the sample was centrifuged (12,000 rpm, 5 min) and the supernatant was retrieved. For the chemical characterization of APE, we employed a hyphenated technique integrating ultra-performance liquid chromatography and quadrupole time-of-flight mass spectrometry (UPLC-Q-TOF/MS).

### 4.3. Analysis of Network Pharmacology and Molecular Docking

The active components of APE were first analyzed for their potential targets using the SwissTargetPrediction database (http://www.swisstargetprediction.ch/, accessed on 3 December 2024). In parallel, NAFLD-linked targets were sourced from GeneCards (https://www.genecards.org, accessed on 3 December 2024) by querying “Nonalcoholic fatty liver disease”. The shared targets between the intervention and the disease were mapped to a Venn diagram via an online bioinformatics portal (http://www.bioinformatics.com.cn/, accessed on 3 December 2024). These overlapping targets subsequently served as the basis for generating a protein–protein interaction (PPI) network on the STRING platform (https://cn.string-db.org/, accessed on 3 December 2024), and the resulting network was rendered and examined using Cytoscape 3.10.2. Further functional characterization through Gene Ontology (GO) and Kyoto Encyclopedia of Genes and Genomes (KEGG) enrichment studies was conducted on the DAVID platform (https://davidbioinformatics.nih.gov/, accessed on 3 December 2024). The findings from these enrichment analyses were then visualized graphically employing the aforementioned online bioinformatics system.

### 4.4. Animals and Grouping

A cohort of sixty male C57BL/6N mice (6–8 weeks old) were acclimatized for one week before being assigned to six experimental cohorts (*n* = 10), comprising: a control (Control) group, an NAFLD model (Model) group, three APE-treated groups at low (APE-L, 100 mg·kg^−1^), medium (APE-M, 200 mg·kg^−1^), and high (APE-H, 400 mg·kg^−1^) doses, alongside a metformin (MET, 280 mg·kg^−1^) treatment group. Mice in both the Control and Model groups were administered an equal volume of distilled water by daily gavage. All experiments concerning animals were ratified by the Ethics Committee of Xinjiang Medical University (Ethic No.: IACUC-20240722-17).

The dosing regimen consisted of a single daily administration of the respective treatments to all groups, and was continued for 12 consecutive weeks. The dietary regimen provided the Control group with a standard diet, whereas all other groups received a high-fat chow, which was sustained for the entire experimental period.

### 4.5. Assessment of Body Weight, Blood Glucose, Serum Lipids, and Liver Function Parameters

Weekly measurements of body weight were obtained for all groups of mice. At week 12, fasting and random blood glucose levels were assessed in tail vein samples via a glucometer (with test strips). To assess glucose homeostasis, the oral glucose tolerance test (OGTT) procedure was carried out. After 12 h of fasting (with free access to water), the mice received an oral gavage of glucose (2 g/kg). Blood samples from the tail vein were used to measure glucose concentrations immediately before (0 min) and at 30, 60, 90, and 120 min after the challenge, allowing for the calculation of the AUC-OGTT. Insulin sensitivity was gauged through the administration of an insulin tolerance test (ITT). Following another 12-h fast, a bolus of human regular insulin (0.5 U/kg) was injected subcutaneously. Glucose measurements were taken from tail blood at baseline (0 min) and 40, 90, and 120 min later, enabling the determination of the AUC-ITT.

Upon completion of the 12-week study, blood was collected from the abdominal aorta of anesthetized (sodium pentobarbital, 35 mg/kg, i.p.) mice and processed by centrifugation at 1000 r/min for 15 min at 4 °C to isolate serum. Serum levels of total cholesterol (TC), triglycerides (TG), high-density lipoprotein cholesterol (HDL-C), low-density lipoprotein cholesterol (LDL-C), aspartate aminotransferase (AST), and alanine aminotransferase (ALT) were measured using respective ELISA kits according to the manufacturers’ instructions.

### 4.6. Histopathological Analysis (H&E Staining)

Liver and colon tissues were processed through a standard protocol involving fixation in 4% paraformaldehyde, sequential dehydration through an ethanol gradient, xylene clearing, and subsequent paraffin embedding. Sections of 5 μm thickness were prepared, mounted on glass slides, and stained with hematoxylin and eosin (H&E) following standard protocols. Histological evaluation and imaging were performed using light microscopy.

### 4.7. Western Blot Analysis

Following homogenization of liver and colon tissues in RIPA lysis buffer, the concentration of total protein was assessed for subsequent analysis employing a BCA protein assay kit. Proteins were separated by SDS-PAGE and transferred to PVDF membranes. The membranes were incubated overnight at 4 °C with the following primary antibodies: Toll-Like Receptor 4 Rabbit pAb (1:500), Phospho-NF-κB p65 (Ser536) Rabbit pAb (1:500), NF-κB p65 Rabbit pAb (1:500), Occludin Rabbit pAb (1:400), ZO-1 tight junction protein Rabbit pAb (1:400) (China Zhengneng Biological Company, Cat# 505258, 310013, 380172, 502601, 164329) (Chengdu, China); MyD88 Monoclonal antibody (1:400), IL-6 Polyclonal antibody (1:500), TNF Alpha Polyclonal antibody (1:400) (Proteintech Group, Inc., Cat# 67969-1-Ig, 26404-1-AP, 17590-1-AP) (Wuhan, China). Protein bands were quantified using ImageJ software v. 2.1.4.7.

### 4.8. 16S rDNA Sequencing of Gut Microbiota

At week 12, fecal samples were collected from mice in the Control, Model, APE-H, and MET groups. Purified from fecal samples by means of the QIAamp DNA Stool Mini Kit (adhering to the supplier’s instructions), the obtained DNA was evaluated for concentration as well as purity on a nucleic acid/protein analyzer. Subsequently, 16S rDNA sequencing of the total bacterial DNA from mice feces was performed by Shanghai Baitique Biotechnology Co., Ltd. (Shanghai, China).

### 4.9. Statistical Analysis

Data analysis was conducted with SPSS 21.0 software. Normality and variance homogeneity in continuous variables were verified, and comparisons were made by one-way ANOVA supplemented with Tukey HSD post hoc testing. Using R version 4.3.0 software, Spearman correlation analysis was performed, and the method used for multiple testing correction was Benjamini–Hochberg (BH). All measured values are reported as mean ± standard deviation (with the notation $\bar{x}$ ± SD). The threshold for statistical significance was established at *p* < 0.05.

## 5. Conclusions

In this study, high-throughput sequencing of gut microbiota in HFD-induced NAFLD mice treated with APE revealed that at the phylum level both interventions modulated key phyla, reducing *Firmicutes* levels and the *Firmicutes/Bacteroidota* ratio concurrently with a rise in *Bacteroidota*. At the genus level, APE and MET markedly decreased the relative abundance of *Desulfovibrionaceae_unclassified*, *Mucispirillum*, and *Oscillibacter*, while increasing the abundance of *Akkermansia*, *Muribaculum*, and *Parabacteroides*. Moreover, APE and MET not only up-regulated colonic tight junction proteins (Occludin and ZO-1) but also down-regulated key mediators of inflammation, including TLR4, MyD88, p-NF-κB p65, the p-NF-κB p65/NF-κB p65 ratio, TNF-α, and IL-6. These findings support a model where a high-fat diet is associated with a cascade of alterations, including an increased abundance of *Desulfovibrio*, elevated intestinal LPS levels, reduced expression of Occludin and ZO-1, and disruption of the intestinal mucosal barrier. Upon binding to intestinal TLR4, LPS activates downstream MyD88/NF-κB p65 signaling, triggering inflammatory cytokine production. This inflammatory response, along with gut barrier dysfunction, is counteracted by APE, which acts by restoring a healthy microbiota, suppressing inflammation, and thereby ameliorating NAFLD.

## Figures and Tables

**Table 1:** Identification results of main components in ethanol extract of the root of *Anacyclus pyrethrum*.

No.	Retention Time (min)	Adduct	Measured *m*/*z*	Theoretical *m*/*z*	ppm	Formula	Mass	Compound	MS/MS Fragments
1	0.94	[M−H]^−^	195.0513	195.0510	1.5	C_6_H_12_O_7_	196.06	Gluconic acid	195.0511; 177.0391; 129.0194; 99.0091; 89.0248; 75.0089; 59.0139
2	1.07	[M−H]^−^	191.0562	191.0556	3.1	C_7_H_12_O_6_	192.06	Quinic acid	191.0548; 127.0387; 93.0334; 85.0284; 59.01284
3	1.21	[M−H]^−^	341.1085	341.1089	−1.2	C_12_H_22_O_11_	342.12	Sucrose	341.1085; 179.0564; 161.0461; 119.0350; 113.0243; 89.0245; 71.0133; 59.0135
4	1.30	[M + FA-H]^−^	475.1320	475.1305	3.2	C_15_H_26_O_14_	430.13	3-Deoxy-9-O-β-D-glucopyranosyl-D-glycero-D-galacto-2-nonulosonic acid	133.0142; 115.0036; 71.0137
5	1.56	[M−H]^−^	191.0199	191.0197	1.0	C_6_H_8_O_7_	192.03	Citric acid	191.0198; 129.0194; 111.0087; 87.0089; 67.0188
6	2.43	[M−H]^−^	191.0198	191.0197	0.5	C_6_H_8_O_7_	192.03	Isocitric acid	191.0194; 129.0191; 111.0089; 87.0087; 67.0189; 57.0344
7	9.29	[M−H]^−^	315.0722	315.0722	0.0	C_13_H_16_O_9_	316.08	Protocatechuic acid-3-O-glucoside	315.0722; 152.0114; 108.0216
8	10.21	[M−H]^−^	205.0716	205.0718	−1.0	C_8_H_14_O_6_	206.08	Methyl quinate	205.0711; 143.0718; 129.0559; 125.8715; 115.0771; 72.9938
9	10.53	[M−H]^−^	439.0560	439.0552	1.8	C_15_H_20_O_13_S	440.06	Glucosyringic acid sulfate	439.0552; 241.0022; 138.9707; 96.9601
10	11.03	[M−H]^−^	359.0984	359.0984	0.0	C_15_H_20_O_10_	360.11	Glucosyringic acid	359.0975; 197.0456; 182.0217; 166.9980; 153.0554; 138.0319; 123.0081; 95.0135
11	11.19	[M−H]^−^	353.0878	353.0878	0.0	C_16_H_18_O_9_	354.10	Neochlorogenic acid	353.0903; 191.0569; 179.0361; 135.0457
12	11.29	[M−H]^−^	447.1149	447.1144	1.1	C_18_H_24_O_13_	448.12	2,6-Dihydroxybenzoic acid 2-O-β-D-apiofuranosyl(1→2)-β-D-glucopyranoside	447.1132; 315.0710; 271.0804; 163.0390; 152.0110; 108.0212
13	11.54	[M−H]^−^	491.1405	491.1406	−0.2	C_20_H_28_O_14_	492.15	/	491.1424; 197.0457; 182.0219; 166.9989; 138.0335
14	12.33	[M−H]^−^	705.1672	705.1672	0.0	C_32_H_34_O_18_	706.17	Dichlorogelignate	705.1674; 513.1045; 339.0502; 321.0397; 229.0138; 191.0564
15	13.17	[M−H]^−^	353.0885	353.0878	2.0	C_16_H_18_O_9_	354.10	Chlorogenic acid	353.0875; 191.0557; 161.0239; 127.0399; 85.0292
16	13.67	[M−H]^−^	353.0872	353.0878	−1.7	C_16_H_18_O_9_	354.10	Cryptochlorogenic acid	/
17	14.95	[M−H]^−^	533.0946	533.0937	1.7	C_24_H_22_O_14_	534.10	2,5-dicaffeoylglucaric acid	533.0943; 371.0609; 209.0299; 191.0195; 179.0349; 129.0187; 85.0292
18	15.86	[M−H]^−^	533.0944	533.0937	1.3	C_24_H_22_O_14_	534.10	3,5-dicaffeoylglucaric acid	533.0925; 371.0640; 209.0303; 191.0197; 85.0305
19	16.40	[M−H]^−^	465.1406	465.1402	0.9	C_22_H_26_O_11_	466.15	/	465.1428; 335.1224; 319.0804; 173.0713; 163.0398; 155.0340; 137.0244
20	17.05	[M−H]^−^	533.0950	533.0937	2.4	C_24_H_22_O_14_	534.10	2,4-dicaffeoylglucaric acid	533.0968; 371.0614; 209.0297; 191.0195; 85.0297
21	17.48	[M−H]^−^	533.0950	533.0937	2.4	C_24_H_22_O_14_	534.10	3,4-dicaffeoylglucaric acid	533.0950; 371.0637; 209.0312; 191.0207
22	18.03	[M−H]^−^	533.0937	533.0937	0.0	C_24_H_22_O_14_	534.10	3,4-dicaffeoylglucaric acid isomer	533.1259; 371.0647; 209.0313; 191.0201; 85.0295
23	18.13	[M−H]^−^	401.1815	401.1817	−0.5	C_19_H_30_O_9_	402.19	(6R)-6-[(3R)-3-(β-D-Glucopyranosyloxy)butyl]-5,5-dimethyl-3-oxo-1-cyclohexene-1-carboxylic acid	401.1840; 221.1186; 177.1286; 101.0242; 71.0133; 59.0137
24	18.85	[M−H]^−^	533.0936	533.0937	−0.2	C_24_H_22_O_14_	534.10	2,4-dicaffeoylglucaric acid isomer	533.0959; 371.0634; 209.0306; 191.0188; 85.0292
25	20.19	[M−H]^−^	515.1220	515.1195	4.9	C_25_H_24_O_12_	516.13	Isochlorogenic acid B	515.1185; 353.0865; 191.0554; 179.0344; 173.0450; 135.0443
26	20.63	[M−H]^−^	515.1216	515.1195	4.1	C_25_H_24_O_12_	516.13	Isochlorogenic acid A	515.1210; 353.0887; 191.0564; 179.0355; 135.0456
27	20.87	[M+H]^+^	595.1663	595.1657	1.0	C_27_H_30_O_15_	594.16	Chrysoeriol 7-O-apiosylglucoside	595.1649; 463.1228; 301.0705; 286.0470
28	21.37	[M−H]^−^	515.1193	515.1195	−0.4	C_25_H_24_O_12_	516.13	Isochlorogenic acid C	515.1182; 353.0863; 191.0549; 179.0341; 173.0447; 135.0444
29	22.64	[M+H]^+^	565.1187	565.1188	−0.2	C_25_H_24_O_15_	564.11	7-[[6-O-(2-Carboxyacetyl)-β-D-glucopyranosyl]oxy]-3,5-dihydroxy-2-(4-hydroxy-3-methoxyphenyl)-4H-1-benzopyran-4-one	565.1183; 479.1182; 317.0654; 302.0424; 285.0392; 257.0437; 153.0190
30	23.54	[M−H]^−^	447.1511	447.1508	0.7	C_19_H_28_O_12_	448.16	Hebitol I	343.1400; 219.0516; 201.0408; 141.0923; 87.0093
31	25.38	[M−H]^−^	581.1869	581.1876	−1.2	C_27_H_34_O_14_	582.19	/	537.1987; 477.1767; 375.1661; 315.1440; 173.0451
32	25.57	[M−H]^−^	581.1896	581.1876	3.4	C_27_H_34_O_14_	582.19	/	581.1871; 537.1987; 477.1767; 375.1665; 315.1449; 201.1135; 173.0458; 151.0929
33	26.21	[M−H]^−^	329.2336	329.2333	0.9	C_18_H_34_O_5_	330.24	9,12,13-Trihydroxy-10-octadecenoic acid	329.2333; 229.1448; 211.1343; 183.1394; 171.1027
34	30.89	[M+H]^+^	278.1547	278.1539	2.9	C_19_H_19_NO	277.15	2-Benzylamino-1-[2]naphthyl-ethanol	278.1531; 157.0655; 128.0616; 105.0694
35	30.99	[M+H]^+^	288.1961	288.1958	1.0	C_18_H_25_NO_2_	287.19	(E,E)-2,4-Decadienamide, N-(p-hydroxyphenethyl)	288.1937; 151.1108; 121.0642; 93.0689
36	31.24	[M+H]^+^	222.1859	222.1852	3.2	C_14_H_23_NO	221.18	Spilanthol	222.1847; 167.1298; 152.1063; 96.0439; 67.0537; 57.0694
37	32.64	[M−H]^−^	293.2119	293.2122	−1.0	C_18_H_30_O_3_	294.22	Hydroxylinolenic acid	293.2117; 275.2014; 235.1702; 183.1032; 171.1031; 121.1025
38	32.73	[M+H]^+^	224.2012	224.2009	1.3	C_14_H_25_NO	223.19	Pellitorine	224.2021; 168.1387; 151.1118; 109.1014; 81.0332; 67.0542
39	32.85	[M−H]^−^	293.2119	293.2122	−1.0	C_18_H_30_O_3_	294.22	13-Hydroxyoctadeca-9,11,15-trienoic acid	293.2102; 275.2000; 223.1318; 195.1377; 183.1373; 171.1009
40	33.26	[M+H]^+^	272.2011	272.2009	0.7	C_18_H_25_NO	271.19	(E,E)-2,4-Tetradecadien-8,10-diynoic acid isobutylamide	272.1995; 1167.1299; 152.1058; 128.0613; 91.0533; 67.0534; 57.0692
41	33.72	[M+H]^+^	316.2265	316.2271	−1.9	C_20_H_29_NO_2_	315.22	(E,E)-N-(p-hydroxyphenethyl)-2,4-Dodecadienamide	316.2282; 179.1436; 161.1328; 121.0646; 91.0538; 81.0331
42	33.98	[M−H]^−^	295.2275	295.2279	−1.4	C_18_H_32_O_3_	296.24	13-Hydroxy-9,11-octadecenoic acid	295.2268; 277.2159; 195.1383; 171.1017
43	34.67	[M−H]^−^	293.2132	293.2122	3.4	C_18_H_30_O_3_	294.22	9-Hydroxyoctadecane-10,12,15-Trienoic acid	293.2112; 249.2207; 195.1379; 179.1071; 167.1081; 113.0973
44	34.80	[M−H]^−^	293.2117	293.2122	−1.7	C_18_H_30_O_3_	294.22	18-Hydroxy-9,11,13-octadecanoic acid	293.2125; 249.2235; 195.1378; 179.1087; 167.1076; 113.0971
45	34.93	[M−H]^−^	293.2117	293.2122	−1.7	C_18_H_30_O_3_	294.22	12-Hydroxy-9,13,15-octadecatrienoic acid	293.2114; 249.2228; 197.1183; 185.1179; 125.0963; 113.0964
46	35.22	[M−H]^−^	293.2118	293.2122	−1.4	C_18_H_30_O_3_	294.22	13-Hydroxy-6,9,11-octadecatrienoic acid	293.2121; 249.2227; 197.1198; 185.1186; 125.0972
47	35.80	[M+H]^+^	252.2322	252.2322	0.0	C_16_H_29_NO	251.22	N-(2-Methylpropyl)-2,8-dodecadienamide	252.2341; 196.1710; 179.1441; 95.0850; 81.0328; 67.0535
48	36.93	[M+H]^+^	278.2482	278.2478	1.4	C_18_H_31_NO	277.24	N-Isobutyl-(2E,4E,8Z)-tetradeca-2,4,8-trienamide	278.2475; 167.1302; 152.1064; 67.0535; 57.0692
49	37.10	[M+H]^+^	266.2481	266.2478	1.1	C_17_H_31_NO	265.24	N-10-Undecen-1-yl-5-hexenamide	266.2497; 196.1713; 179.1448; 95.0859; 81.0337
50	38.68	[M−H]^−^	277.2171	277.2173	−0.7	C_18_H_30_O_2_	278.22	Linolenic acid	277.2167; 233.2293; 134.8945
51	38.88	[M+H]^+^	280.2642	280.2635	2.5	C_18_H_33_NO	279.26	Linoleamide	280.2632; 224.2007; 109.1005; 98.0589; 81.0691; 29.0691; 57.0691
52	40.28	[M+H]^+^	609.2713	609.2694	3.1	C_34_H_40_O_10_	608.26	Scortechinone C	609.2710; 591.2599; 558.2354; 550.2581; 531.2379; 515.2456
53	40.41	[M−H]^−^	279.2332	279.2330	0.7	C_18_H_32_O_2_	280.24	Linoleic acid	279.2317; 261.2241
54	41.05	[M+H]^+^	609.2716	609.2694	3.6	C_34_H_40_O_10_	608.26	Scortechinone M	609.2741; 591.2630; 559.2369; 531.2413; 515.2467; 485.2358
55	41.68	[M+H]^+^	593.2749	593.2745	0.7	C_34_H_40_O_9_	592.26	Scortechinone F	593.2768; 533.2559; 460.2248

**Table 2:** Repeated measures ANOVA of body weight in each group of mice after 12 weeks of intervention.

Effect		Sum of Squares (SS)	Degrees of Freedom (dfs)	F-Value	*p*-Value	Partial η^2^
within-subjects effect						
	time	21,877.904	2.538	591.445	0.000	0.916
	Time × group	2377.754	12.690	12.856	0.000	0.543
	error	1997.493	137.055			
between-subjects effect						
	group	4981.316	5	13.189	0.000	0.550
	error	4078.884	54			

## Data Availability

The raw data supporting the conclusions of this article will be made available by the authors without undue reservation.

## Abbreviations

The following abbreviations are used in this manuscript:

NAFLD	Nonalcoholic fatty liver disease
APE	*Anacyclus pyrethrum* root ethanol extract
OGTT	Oral glucose tolerance test
ITT	Insulin tolerance test
TC	Total cholesterol
TG	Triglycerides
HDL-C	High-density lipoprotein cholesterol
LDL-C	Low-density lipoprotein cholesterol
AST	Aspartate aminotransferase
ALT	Alanine aminotransferase
H&E	Histopathological analysis
APE-L	APE-treated groups at low
APE-M	APE-treated groups at medium
APE-H	APE-treated groups at high
MET	Metformin
